# The cost effectiveness of NHS physiotherapy support for occupational health (OH) services

**DOI:** 10.1186/1471-2474-13-29

**Published:** 2012-02-23

**Authors:** Ceri J Phillips, Rhiannon Phillips (nee Buck), Chris J Main, Paul J Watson, Shân Davies, Angela Farr, Christie Harper, Gareth Noble, Mansel Aylward, Julie Packman, Matt Downton, Janine Hale

**Affiliations:** 1 Swansea Centre for Health Economics, College of Human and Health Sciences Swansea University, Swansea, UK; 2 Mental Health Research and Development Unit, School for Health, University of Bath, Bath, UK; 3 Arthritis Research Campaign National Primary Care Centre, Primary Care Sciences, Keele University, Keele, UK; 4 Department of Health Sciences, University of Leicester, Leicester, UK; 5 Centre for Psychosocial and Disability Research, Cardiff University, Cardiff, UK; 6 Department for Public Health and Health Professions, Welsh Government, Cardiff, UK; 7 Department of the First Minister and Cabinet, Welsh Government, Cardiff, UK

**Keywords:** Musculoskeletal conditions, Quality of life, Cost-effectiveness analysis, Physical therapy, Occupational health

## Abstract

**Background:**

Musculoskeletal pain is detrimental to quality of life (QOL) and disruptive to activities of daily living. It also places a major economic burden on healthcare systems and wider society. In 2006, the Welsh Assembly Government (WAG) established a three tiered self-referral Occupational Health Physiotherapy Pilot Project (OHPPP) comprising: 1.) telephone advice and triage, 2.) face-to-face physiotherapy assessment and treatment if required, and 3.) workplace assessment and a return-to-work facilitation package as appropriate. This study aimed to evaluate the feasibility and cost-effectiveness of the pilot service.

**Methods:**

A pragmatic cohort study was undertaken, with all OHPPP service users between September 2008 and February 2009 being invited to participate. Participants were assessed on clinical status, yellow flags, sickness absence and work performance at baseline, after treatment and at 3 month follow up. Cost-effectiveness was evaluated from both top-down and bottom-up perspectives and cost per Quality Adjusted Life Year (cost/QALY) was calculated. The cost-effectiveness analysis assessed the increase in service cost that would be necessary before the cost-effectiveness of the service was compromised.

**Results:**

A total of 515 patients completed questionnaires at baseline. Of these, 486 were referred for face to face assessment with a physiotherapist and were included in the analysis for the current study. 264 (54.3%) and 199 (40.9%) were retained at end of treatment and 3 month follow up respectively. An improvement was observed at follow up in all the clinical outcomes assessed, as well as a reduction in healthcare resource usage and sickness absence, and improvement in self-reported work performance. Multivariate regression indicated that baseline and current physical health were associated with work-related outcomes at follow up. The costs of the service were £194-£360 per service user depending on the method used, and the health gains contributed to a cost/QALY of £1386-£7760, which would represent value for money according to current UK thresholds. Sensitivity analyses demonstrated that the service would remain cost effective until the service costs were increased to 160% per user.

**Conclusions:**

This pragmatic evaluation of the OHPPP indicated that it was likely to be feasible in terms of service usage and could potentially be cost effective in terms of QALYs. Further, the study confirmed that improving physical health status for musculoskeletal pain patients is important in reducing problems with work capacity and related costs. This study suggests that this type of service could be potentially be useful in reducing the burden of pain and should be further investigated, ideally via randomised controlled trials assessing effectiveness and cost-effectiveness.

## Background

The economic impact of pain is greater than most other health conditions [1,2] due to its effects on rates of absenteeism, reduced productivity and risk of leaving the labour market. The indirect (productivity) cost of back pain in the UK was estimated to be between £5 billion (€6.7 billion) and £10.7 billion (€14.4 billion) in 1998, depending on the approach employed [1]. The average cost of sickness absence to industry was estimated at £666 per employee per year in 2008 [3]. Of this, musculoskeletal pain may represent as much as 49% of the total cost of sickness absence lasting longer than 3 days [4].

A recent poll of members of the Chartered Institute for Personnel Development found that after seasonal coughs and colds, musculoskeletal conditions were the foremost reason for short-term work absence in manual workers and the second most common reason for long term absences [3]. These data are supported by the findings of a prospective study of work absence in a large working population [5].

It has been estimated that as much as 77% of lost productivity associated with pain relates to reduced performance rather than work absence [6]. In addition, the odds of quitting one's job because of ill health have been shown to be seven times higher among people with chronic pain problems than those without [7]. In a recent survey conducted in Wales, nearly 43% of employees with a health condition in the previous 12 months, which they believed was caused or made worse by work, identified a musculoskeletal pain condition [8]. Any condition caused or made worse by work falls under the remit of Occupational Health (OH) services.

In 2005, the Welsh Assembly Government (WAG) commissioned a mapping exercise to investigate the level of OH activity across Wales [9]. One of the key recommendations of the report was to explore opportunities to support OH health care professionals by provision of physiotherapists to cover more of the musculoskeletal workload. It should be noted that in the United Kingdom, there is only one OH specialist employed in every 35 medium-sized businesses (defined as between 50 and 250 employees) and only 1 in every 1506 small businesses (less than 50 employees). The National Health Service (NHS) is therefore the only OH service available for many workers with musculoskeletal pain [10].

There is evidence to support the effectiveness of comprehensive and integrated return to work packages [11-13]. However, there is little evidence concerning the practical implementation of this evidence into health policy and clinical practice and few assessments of whether such an approach is cost effective and there is a dearth of studies investigating the cost effectiveness of physiotherapy for musculoskeletal pain

This paper represents the establishment of a pilot project of a physiotherapy service provided by three NHS Trusts in Wales--Hywel Dda, Gwent and North West Wales.^a ^The self-referral service was made available to public, private and third sector organisations and included 47 small, medium and large employers representing a total of approximately 28,000 employees. The aim of this paper is to report on the evaluation of the programme pilot, focussing on feasibility and cost-effectiveness.

## Methods

### Development of the service

The Occupational Health Physiotherapy Project Pilot (OHPPP) service was provided in three stages:

• Physiotherapist telephone advice and triage to provide rapid, easily accessible advice and signposting to relevant services;

• Physiotherapist face-to-face assessment and treatment, arranged via telephone advice and triage if deemed appropriate, to alleviate signs and symptoms of musculoskeletal disorders (MSDs) and provided in hospital, workplace, satellite clinics and other settings;

• Workplace assessment to provide essential information to enable early return to work and resolution of a slow-to-recover problem or a recurrent MSD, along with assisting in overcoming fears associated with return to work and informing the process of negotiating and formulating a return to work package.

All employees of the organisations that had signed up to take part in the scheme were informed of the OHPPP service, primarily via leaflets that were provided to those on sick leave and those in work. The leaflet outlined the nature of the scheme and contained contact details for the telephone advice line including operational hours. The line was staffed by a qualified senior physiotherapist with clinical experience in musculoskeletal conditions. Patients provided with a face-to-face assessment and workplace assessment were seen by a senior musculoskeletal physiotherapist trained in Occupational Health and Ergonomics. A job specification was set for physiotherapists working alongside the senior physiotherapists on the project, who were clinical specialists, extended scope practitioners, and some lower grade staff, depending on the nature of clinical input being provided. Training was provided for all staff involved at each of the pilot sites covering aspects of Occupational Health, work and ergonomics. The face-to-face assessment and treatment part of the service was located within NHS hospitals and in satellite clinics within the relevant NHS Trust areas.

### Design and participants

A cohort study was undertaken, with all employees contacting the OHPPP service between September 2008 and February 2009 being invited to participate. At its inception it was estimated that approximately 150 people a month would be expected to use the service and it was estimated that of the users of the scheme:

• 64% would receive face to face assessment and treatment

• 25% would be managed by advice alone

• 11% would be inappropriate

• 36% would receive work place assessments

**Figure 1:**
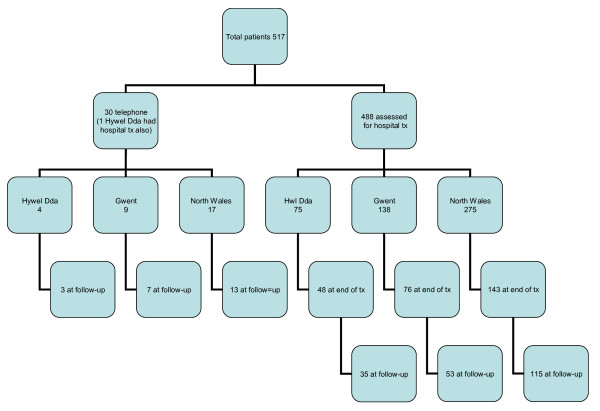
**OHPPP participant flow diagram**.

It was therefore expected that roughly 96 people a month would be referred on to further stages of the intervention and the study was therefore set up to follow consecutive referrals over a 12 week period to provide adequate data for the evaluation of the service. However, it became apparent that uptake of the scheme was not as had been anticipated and uptake was 54% of what had been anticipated. All employees that consented to participate were given questionnaires at entry to the scheme (baseline), at end-of-treatment and at 3-month follow-up, with the exception of employees that were provided with telephone advice only who were provided with questionnaires at entry and 3 month follow-up only. Participant flow is shown in Figure 1.

515 questionnaires were completed by service users, of whom 29 received telephone advice only and 486 (94%) were given a face to face hospital based and workplace assessment if required. Due to the small number of participants receiving telephone advice only, analysis on pre- to post- change in outcome variables was restricted to those who had received face to face treatment (n = 486). 264 (54.3%) were retained at end of treatment and 199 (40.9%) and 3 month follow up.

## Measures

The questionnaires comprised a series of validated instruments designed to assess the clinical, economic and quality of life (QOL) effects of the scheme. Measures of psychosocial risk factors, known as 'yellow flags' such as fear, avoidance and catatrophizing were also included. The questionnaire set included the following measures:

• Örebro Musculoskeletal Pain Questionnaire (ÖMPQ) (at baseline only) [14]

• Pain Catastrophising Scale [15]

• Fear Avoidance Beliefs Questionnaire (including work and physical activity subscales) [16]

• Location specific pain measures [17-20]

- Roland Morris

- DASH (arm, shoulder and hand)

- Neck Disability

- Lower extremity

• Health related quality of life measures [21-23]

- EQ-5D (quality of life)

- SF-12 (including mental and physical health subscales)

- GHQ (psychological distress)

### Statistical analysis

Statistical analysis was carried out using PASW Statistics Version 18. A descriptive analysis of the baseline characteristics and demographic profile of the sample was carried out, and comparisons between those who were retained or lost to follow up was carried out using ANOVA and Cramer's V test as applicable to assess any potential systematic bias in drop out. Changes in the Clinical, yellow flags and work-related variables between baseline and end of treatment and follow up were assessed using t-tests.

Due to the significant economic costs of the impact of pain on work performance and absence, multivariate regression analysis was carried out to assess whether current health status and yellow flags were independently associated with these outcomes at end of treatment and follow up, adjusting for baseline scores on these scales and demographics. Demographic variables (age and sex), baseline and current clinical variables and yellow flags (SF12 PCS & MCS, EQ-5D, GHQ, Pain VAS, fear and avoidance beliefs) were entered in to the models simultaneously using the 'Enter' command in PASW. Missing data was excluded listwise.

### Cost- effectiveness analysis

The cost-effectiveness of interventions is generally assessed by way of an incremental cost effectiveness ratio (ICER) versus usual standard care. An ICER takes into account the costs and benefits of the two comparative interventions. Benefits are often measured using Quality Adjusted Life Years (QALYs), which take into account both length and quality of life. In so doing, the QALY enables comparison across a wide range of interventions with different outcomes. QALYs are utilised in the cost-effectiveness analyses assessed by the National Institute of Health and Clinical Excellence (NICE). Cost-effectiveness is determined according to whether the cost/QALY is within acceptable limits according to the funding/decision making body. For NICE, the willingness-to-pay threshold is often understood to be around £20K-£30K/QALY, where interventions with an ICER which exceeds this threshold, deemed not to a cost-effective use of NHS resources. The cost-effectiveness of OHPPP was assessed from the perspective of the UK NHS and therefore indirect costs have not been included. Given that the participants also acted as their own controls, the comparator was basically what would be usual care and practice. The cost-effectiveness was assessed over a period of 12 months, based on assumptions regarding duration of benefits. Cost data were provided by WAG--based on budgetary and expenditure records--and from published unit cost data. Effects were derived from the findings relating to clinical and QOL impacts of the scheme. The costs associated with OHPPP were derived from both top-down and bottom-up perspectives. The top down approach was employed to relate the agreed budgeted expenditure to each service provided by the NHS sites, while published unit cost data was used in conjunction with resource usage to derive cost estimates of specific services from a bottom-up perspective. A series of sensitivity analyses were undertaken to assess the effects of variations in costs and outcomes and their impact on the findings of the evaluation.

## Results

A summary of the characteristics of the 486 participants at baseline is provided in Table 1. Responders for all three assessments were compared to those lost to follow up after baseline or end of treatment were compared on age, duration of pain complaint, SF12 physical and mental health sores, GHQ, EQ5D, and pain. Cramer's V tests for the categorical variables indicated that those lost to follow up did not differ from responders in gender, income band, work status over the last 6 months, or pain site. Baseline scores on the outcome variables are provided in Table 2. The only differences found between completers and non-completers at follow up were in age (F = 9.53, df 2, 473, p < 0.001) and EQ-5D score (F = 3.51, df 2, 479, p < 0.05). Post-hoc Bonferroni tests indicated that those completing all three assessments had higher EQ5D scores than those who completed baseline and end of treatment assessments, and those completing all three assessments were older than those completing only baseline or baseline and post treatment assessments.

**Table 1:** Baseline characteristics of participants referred for face-to-face assessments (N = 486)

Variable		N (%)
Gender	Male	175 (36)
	Female	306 (63)
	*Missing *	*5 (1)*
Job type	Managerial/supervisory	79 (16.3)
	Non-manual	284 (58.4)
	Manual	121 (24.9)
	*Missing*	*2 (0.4)*
Work situation - past 6 months	Usual hours and duties	344 (72.3)
	Usual hours but not usual duties	44 (9.2)
	Usual duties but not usual hours	11 (2.3)
	Usual hours but help needed	69 (14.5)
	Not worked during treatment	8 (1.7)
	*Missing*	*10 (2.1)*
Pain site	Back	
	Neck	
	Arms, shoulders, and/or hands	
	Lower extremities	
		**Mean (SD)**
Age		43.10 (10.45)
Pain duration (months)		56.12 (91.1)

**Table 2:** Mean and standard deviation of outcome variables at baseline, end of treatment and 3 month follow up

Variable	Baseline	End of treatment	Follow up
Clinical			
Pain intensity VAS	10.54 (9.4)	7.42 (8.5)***	6.91 (9.4)***
GHQ	12.95 (6.1)	9.76 (4.6)***	10.11 (5.7)***
SF-12 mental health	52.32 (11.2)	55.52 (8.5)***	55.81 (8.5)***
SF-12 physical health	42.18 (8.5)	48.86 (7.2)***	50.97 (7.5)***
EQ-5D	0.66 (0.2)	0.82 (0.2)***	0.82 (0.2)***
Yellow flags			
Pain Catastrophizing	10.54 (9.41)	7.42 (8.54)***	6.91 (9.4)***
Fear and avoidance - work	9.79 (9.5)	7.92 (8.3)***	8.18 (8.6)***
Fear and avoidance - physical activity	11.81 (6.4)	8.31 (6.5)***	7.63 (6.0)***
Work-related			
Sickness absence	4.6 (12.6)	2.82 (11.4)*	1.45 (9.7)*
Work performance	75.9 (19.6)	82.1 (16.2)***	87.8 (13.2)***

Statistically significant difference from baseline scores at end of treatment and follow-up using paired samples t-tests are denoted by *p < 0.05, **p < 0.01, ***p < 0.001

### Change in clinical, psychosocial and work-related variables

Means and standard deviations for the clinical, psychosocial and work related variables at baseline, end of treatment and follow up along with significant differences from baseline are shown in Table 2. Statistically significant changes in the expected direction were observed for all of these target variables.

Statistically significant models emerged in multivariate regression analysis for all four sets of analysis; days sickness absence at end of treatment (F = 3.06, df 17, 222, p < 0.001, Adjusted R^2 ^= 12.6%) and at 3 month follow up (F = 3.31, df 17, 151, p < 0.001, Adjusted R^2 ^= 18.9%), and work performance at end of treatment (F = 7.40, df 17, 220, p < 0.001, Adjusted R^2 ^= 31.5%) and 3 month follow up (F = 4.43, df 17, 154, p < 0.001, Adjusted R^2 ^= 25.4%). Beta values and 95% Confidence Intervals for the variables entered in to the models are provided in Table 3.

**Table 3:** Multivariate regression for associations with sickness absence and work performance at end of treatment and follow up

	End of treatment		3 month follow up	
	Β	95% CIs	β	95% CI
**Days sickness absence (last 6 months)**				
**Baseline**				
Age	-0.12*	**-0.24, -0.01**	-0.05	-0.19, 0.09
Gender	-0.72	-3.00, 1.56	1.70	-1.12, 4.52
SF-12 PCS	-0.27**	**-0.44, -0.10**	-0.05	-0.24, 0.15
SF-12 MCS	-0.05	-0.22, 0.14	-0.17	-0.39, 0.04
EQ-5D	1.66	-5.46, 8.77	1.07	-7.43, 9.57
GHQ	-0.18	-0.49, 0.13	0.05	-0.32, 0.42
Pain VAS	-0.11	-0.28, 0.07	-0.21*	**-0.41, -0.01**
OREBRO	0.08*	**0.001, 0.15**	-0.06	-0.15, 0.03
Fear and Avoidance Beliefs - Work	0.11	-0.37, 0.04	-0.16	-0.34, 0.03
Fear and Avoidance Beliefs - Physical Activity	-0.17	-0.25, 0.14	0.10	-0.15, 0.35
**Current status**				
SF-12 PCS	-0.05	-0.25, 0.14	-0.35**	**-0.58, -0.11**
SF-12 MCS	-0.08	-0.28, 0.13	0.007	-0.24, 0.25
EQ-5D	4.18	-4.28, 12.64	-3.23	-12.18, 5.71
GHQ	0.17	-0.18, 0.51	0.92	-0.29, 0.47
Pain VAS	-0.06	-0.23, 0.11	0.91	-0.10, 0.28
Fear and Avoidance Beliefs - Work	-0.04	-0.22, 0.15	0.18	-0.02, 0.38
Fear and Avoidance Beliefs - Physical Activity	0.18	-0.05, 0.41	0.13	-0.13, 0.41
**Work performance (last 30 days)**				
**Baseline**				
Age	0.04	-0.13, 0.21	-0.12	-0.30, 0.07
Gender	2.27	-1.24, 5.77	-0.97	-4.65, 2.71
SF-12 PCS	0.32*	**0.06, 0.58**	0.03	-0.22, 0.28
SF-12 MCS	0.23	-0.05, 0.50	0.01	-0.27, 0.30
EQ-5D	4.84	-6.05, 15.73	-4.38	-15.43, 6.67
GHQ	0.07	-0.41, 0.55	0.27	-0.21, 0.76
Pain VAS	0.08	-0.19, 0.35	-0.19	-0.46, 0.07
OREBRO	0.05	-0.06, 0.16	-0.05	-0.16, 0.07
Fear and Avoidance Beliefs - Work	-0.10	-0.37, 0.16	0.09	-0.16, 0.33
Fear and Avoidance Beliefs - Physical Activity	0.16	-0.16, 0.47	0.05	-0.27, 0.38
**Current status**				
SF-12 PCS	0.85***	**0.55, 1.15**	0.53**	**0.22, 0.84**
SF-12 MCS	0.27	-0.04, 0.59	0.25	-0.07, 0.58
EQ-5D	-5.43	-18.43, 7.58	4.03	-8.08, 16.14
GHQ	-0.52	-1.04, 0.02	-0.44	-0.93, 0.06
Pain VAS	0.09	-0.17, 0.35	-0.02	-0.27, 0.24
Fear and Avoidance Beliefs - Work	-0.15	-0.43, 0.13	-0.20	-0.46, 0.07
Fear and Avoidance Beliefs - Physical Activity	-0.25	-0.60, 0.10	0.12	-0.23, 0.46

NB: Values shown to 2 decimal places. * *p *< 0.05, **p < 0.01, ***p < 0.001

### Healthcare utilization

Of the 197 respondents who completed questionnaires at all time points, 96 (48.7%) reported that they had utilised healthcare services at baseline compared with 24 (12.2%) at end of treatment and 23 (11.7%) at follow up (p < 0.001). There was also a significant reduction in summary score for pain between baseline and end of treatment (p < 0.001) and at follow-up (p < 0.001).

### Service uptake rates and cost of service provision

The agreed expenditure for the three sites was based on estimates of demand during the establishment of the scheme, which were that approximately 150 people would contact the service each month with a conversion rate of around 64% to face-to-face contact. The actual demand for the scheme was only 54% of what had been anticipated, whilst the conversion rate of telephone consults into face to face assessments was 94%. All the service users who received a workplace assessment had first received both telephone advice and a face-to-face assessment.

Based on contact times provided during discussions with service providers it has been estimated that the cost per service user contact hour would be in the region of £46. This compared with the published unit cost of £40 per service user hour (including training of staff involved), shown in Table 4.

**Table 4:** Costs of Service Provision

	Staff time (hrs)	Cost based on published hourly rate (£)*	Cost based on budgeted expenditure (£)***	Actual Cost (£)
Telephone	0.75	30	34.66	64.56
Face to face	4	160	184.87	344.31
Workplace	6 (inc travel)	273	277.30	516.47
Unit cost per hour		40**	46.22	86.08
Unit cost per service user		168.00	194.00	360.00

*Curtis L. Unit costs of health and social care (p.195) 2010. http://www.pssru.ac.uk/pdf/uc/uc2010/uc2010.pdf

**including qualifications

***Based on Welsh Assembly Government estimates of expenditure

While the original budget estimates were a considerable over-estimate in terms of demand, the conversion rates to face to face consultations is likely to have limited the discrepancy between estimated and actual costs of delivering the service. The calculated unit cost per service user hour amounted to £86--1.87 times the estimated amount. Based on the assumptions relating to cost per hour, it is estimated that the cost per user receiving the OHPPP service amounted to between £194 (when the bottom-up approach was employed) and £360 (based on the top-down approach from WAG expenditure).

### Cost-effectiveness analysis

The extent of the benefits highlighted in the previous section indicates the relative success of the scheme in relation to economic effects, clinical benefits and QOL gains. For example, the gain in health status between baseline and follow-up (3 months after end of treatment) was 0.14 as measured by EQ-5D, which translates to a gain of 0.047 QALYs, if the beneficial effect was terminated at follow-up. If it is assumed that the beneficial effect was sustained for a longer period of time, (e.g. 12 months), then the QALY gain would amount to 0.14 per employee.

In this case, the estimated cost/QALY would be between £1386 (when cost = £194 and QALY gain = 0.14) and £7660 (when cost = £360 and QALY gain = 0.047) and would be well within the bounds of cost/QALY estimates considered--by the National Institute of Health and Clinical Excellence (NICE) and other assessment agencies--to represent value for money. In order for the cost/QALY to be equivalent to NICE's cost-effectiveness threshold of £20,000, there would have to be an increase of 160% in the cost per user, using highest cost estimate or a reduction of 62% in the QALY gained, using the lower utility estimate derived from the EQ5D responses. As an alternative approach, if one assumes that the value society place on a QALY is equivalent to £25,000, then the net benefit (i.e. total benefits minus total costs) generated by OHPPP is between £3,140 and £13,806 per employee participant.

## Discussion

### Rationale for the scheme

There is good evidence that an early intervention approach to the management of musculoskeletal disorders can prevent work loss in those who are symptomatic and reduce the time to return to work for those who absent from work [24-26]. There is also increasing evidence that early intervention approaches, particularly those that address return to work can be cost effective for employers, health purchasers and providers of wage replacement benefits [27-30].

Numerous systematic and narrative reviews have concluded that early treatment for musculoskeletal pain problems can reduce work-loss and improve the patient's chances of rehabilitation and sustained retention in work [31]. The evidence that physical therapy interventions alone are effective in returning people to work is equivocal [32]. One previous study demonstrated early referral to physiotherapy was more effective than usual care [33]. However, the provision of appropriate workplace assessments and the incorporation of recommendations from such an assessment by way of modified duties may help the worker return to work sooner [12,34,35].

Providing active rehabilitation, rather than simply the provision of symptomatic relief (analgesia and manual therapies), is an effective way of speeding up return to work and reducing work loss in the longer term [36]. The aim of the current intervention was to provide symptomatic relief where required, but to focus on active rehabilitation to assist the person to return to work. The basic elements of an active rehabilitation programme include; advice on activity management including work, graded physical exercise, and early resumption of avoided or ceased activities using a cognitive behavioural approach [37]. Numerous reviews of the literature [35,36,38-40] have indicated that this is the most effective and cost-effective way to help workers with musculoskeletal pain problems from a variety of diagnoses. Guidance and recommendations from NICE relating to the management of long-term sickness absence and incapacity reflect such evidence [41].

### Service uptake

Uptake rates for this service were lower than expected, with around half of the anticipated number of service users contacting OHPP. However, the vast majority of people contacting the service were referred for face-to-face treatment. Uptake for the workplace assessments, however, was also lower than expected. Approximately 2-3% of the employees at participating organisations contacted the service over the 8 month period when the evaluation was being conducted. The vast majority of people with acute musculoskeletal pain (approximately 98%) spontaneously recover relatively quickly [32,38], and therefore the rate of contact with the OHPP service may reflect this. This may have meant that those with more minor or self-limiting complaints did not require the telephone advice to the extent that had been anticipated. Indeed, the average pain duration of participants in this study was 56 months indicating that they had persistent problems.

The Welsh Backs initiative, promoting awareness of recommendations for the self-management of musculoskeletal pain for both members of the public and health professionals, had been taking place around the time that this service was introduced and therefore this may also have influenced the way in which the OHPP service was used. It is also possible that telephone advice alone could have been sufficient for some of the service users referred for face to face contact. The lack of referrals for workplace assessments could have been due to a number of reasons, such as the availability of OH services in the participating workplaces and possibly reluctance on the part of the clinicians and service users to take the management of what they perceived to be a clinical problem in to a workplace setting. It is possible that further education and training would be required to increase uptake of this component of the service.

In summary, these findings suggested that in terms of adoption and implementation of the service, the face-to-face hospital based contact was most successful, with little demand for telephone advice alone or workplace assessments. The reasons for this would need further investigation if the service were to be rolled out.

### Change in outcomes and cost-effectiveness

As this was a cohort study and no control group was included, it is not possible to conclude to what extent to which changes observed were due to spontaneous recovery as opposed to being a result of the intervention. Nonetheless, statistically significant improvements were observed in all the outcomes shown in Table 2 between the baseline and follow up assessments. Current health status had the strongest association with work-related variables and association was stronger for work performance than for absence, which was similar to findings from a previous study conducted by some of the authors [42,43], and highlights the importance of managing MS pain effectively.

Furthermore, cost-effectiveness analyses indicate that this service could potentially be provided at an acceptable cost for the level of benefit yielded. The analysis indicated that the service would continue to be cost-effective until the cost per user increased by 160%. Furthermore, it would be anticipated that once such a service becomes established and the uptake increases the cost per case would reduce.

It should be noted that the wider societal effects associated with reduced sickness benefits costs, reduced costs to employers attributed to improved production and reduced absence rates were not included in the current analysis. Sickness absence and presenteeism in particular are associated with significant economic costs [3,4]. However, we did examine associations with work performance and absence at follow up and end of treatment within the cohort using multivariate regression analysis. This indicated that physical health status was particularly important in understanding impaired work capacity at follow up. Current physical health status (independently of baseline physical health) had the strongest association with work performance and absence at 3 month follow up, confirming the importance of timely and effective management of musculoskeletal pain in reducing its burden.

### Methodological issues

First and foremost, it must be stressed that this evaluation employed a pragmatic cohort design and was not based on a Randomised Controlled Trial (RCT). This means that it is not possible to state that the benefits seen were due to the intervention employed (the OHPPP scheme). Nonetheless, a cohort design is a pragmatic approach to initial evaluation of clinical practice, and this study indicate that there was change in the relevant variables in the expected direction and that it could potentially be cost-effective. This suggests that further investigation using an RCT is warranted

Despite regular and on-going marketing of the scheme, the level of engagement by employers and service users was considerably less than had been anticipated when the scheme was being established. This had an impact on participant numbers resulting in the extension of the recruitment period from 12 weeks to 7 months. Furthermore, many service users failed to return follow-up questionnaires. These issues are important in terms of the representativeness of the sample and ability to generalise from the findings and improving engagement and minimising attrition are important issues for further trials of such interventions.

## Conclusions

This study demonstrated that that the OHPP is feasible and has the potential to represent good value for money. It is the first study to present cost effectiveness data from physiotherapy intervention involving a partnership between the NHS and employers, and it is well within the thresholds considered to indicate relative cost-effectiveness for the UK National Health Service (NHS). Despite the methodological issues, there are grounds for considering that OHPPP is feasible as a service and has met the objectives posed for the pilot scheme. Improvement was observed across a range of validated clinical measures in the quality of life of service users, and in work related outcomes (absence and performance). These results strongly suggest that the scheme has potential to be beneficial and cost effective and further trial(s) of such initiatives that include appropriate control groups would be warranted with an assessment of wider societal costs.

## Endnotes

^a^These have now been reorganised into Hywel Dda Local Health Board, Aneurin Bevan Health Board and Betsi Cadwalader University Local Health Board

## Competing interests

The authors declare that they have no competing interests.

## Authors' contributions

CP was the PI on the study and, along with RB, CM, PW, SD, GN and MA as co-applicants, designed the investigation and drafted the study report and subsequent paper. AF was responsible for data collection and was involved in the drafting of study report. JR, MD and JH commissioned the study, were responsible for facilitating data collection and have been involved in drafting the report and have given approval of the paper submitted. CH was involved with drafting/editing the paper. All authors read and approved the final manuscript.

## Funding

The academic institutions received an educational grant from the Welsh Assembly Government to conduct this pilot study.

## Pre-publication history

The pre-publication history for this paper can be accessed here:

http://www.biomedcentral.com/1471-2474/13/29/prepub
